# The Dissociation between Polarity, Semantic Orientation, and Emotional Tone as an Early Indicator of Cognitive Impairment

**DOI:** 10.3389/fncom.2016.00095

**Published:** 2016-09-14

**Authors:** Susana A. Arias Tapia, Rafael Martínez-Tomás, Héctor F. Gómez, Víctor Hernández del Salto, Javier Sánchez Guerrero, J. A. Mocha-Bonilla, José Barbosa Corbacho, Azizudin Khan, Veronica Chicaiza Redin

**Affiliations:** ^1^Facultad de Ciencias Humanas y de la Educación, Universidad Técnica de AmbatoAmbato, Ecuador; ^2^Departamento de Filosofia, Universidad Técnica Particular de LojaLoja, Ecuador; ^3^Departamento de Inteligencia Artificial, Universidad Nacional de Educación a DistanciaMadrid, España; ^4^Psychophysiology Laboratory, Department of Humanities and Social Sciences, Indian Institute of Technology BombayMumbai, India

**Keywords:** cognitive impairment, emotion, semantic polarity, tonality

## Abstract

The present study aims to identify early cognitive impairment through the efficient use of therapies that can improve the quality of daily life and prevent disease progress. We propose a methodology based on the hypothesis that the dissociation between oral semantic expression and the physical expressions, facial gestures, or emotions transmitted in a person's tone of voice is a possible indicator of cognitive impairment. Experiments were carried out with phrases, analyzing the semantics of the message, and the tone of the voice of patients through unstructured interviews in healthy people and patients at an early Alzheimer's stage. The results show that the dissociation in cognitive impairment was an effective indicator, arising from patterns of inconsistency between the analyzed elements. Although the results of our study are encouraging, we believe that further studies are necessary to confirm that this dissociation is a probable indicator of cognitive impairment.

## Introduction

Scientific research related to geriatric health problems is gaining importance in our global society. The World Federation for Mental Health states that it is important to study adults over 60 years of age because their population will exceed 2000 million by 2050 (Evans-Lacko et al., 2016). The elderly population is known to suffer from mental illness that is characterized by depression and anxiety. However, the major concern of elderly people is affordable mental health care. The prevention of mental illness includes avoiding isolation. Conversations with family members and the use of social networks help to prevent depression that is caused by isolation, which in turn produces irreversible changes in mental health (Haritou et al., 2016). Older adults may also experience difficulties in performing activities that they used to do fairly easily, which may alter behavior due to stress. Psychologists are mainly concerned with the management of behavioral problems in older people, finding it necessary to create a safe environment to monitor patients using automatic or semiautomatic procedure. A patient with cerebral lesions may lack the ability to emotionally register pain and suffering. Based on this view, the communication of cerebral neuronal networks determines whether pain, disenchantment, or depression is presented (Evans-Lacko et al., 2016). Previous studies have evaluated behavioral changes in the elderly through the analysis of written text (Cole-Lewis and Kershaw, 2010). In addition, voice analysis can be used to indicate signs of disease in the elderly (Naranjo et al., 2015). Some people who have experienced high levels of stress or cognitive impairment tend to temporarily dissociate the verbal conversation from the non-verbal (Howel and Itzkowitz, 2016). In the present study, we investigated whether the text of an interview and tone of voice were related, which could indicate whether relatives or caregivers should be alerted to the behavioral changes of the patients.

The objective of this research was to define a procedure for the diagnosis of cognitive impairment (CI) that may be associated with early stages of Alzheimer's disease (AD) based on the analysis of emotional behavior. Such a procedure could be used for routine clinical evaluation or screening before the use of more expensive and selective tests that accurately confirm or rule out the presence of disease. Moreover, this approach allows us to study the dissociation between referential variables, which has not been performed in other AD studies (Cole-Lewis and Kershaw, 2010; Naranjo et al., 2015; Evans-Lacko et al., 2016; Haritou et al., 2016) We used an economic and simple filter method that possibly could be integrated into telecare services, which would give an early warning similar to other more expensive diagnostic instruments and help to increase the confidence in the diagnostic results.

To evaluate the dissociation between the text and tone of an interview, we selected two characteristics that are associated with the semantics of a text, i.e., polarity and semantic orientation. Polarity refers to the positive or negative interpretation that an expert makes regarding a phrase or conversation. This interpretation is based on specialized dictionaries. Semantic orientation refers to the positive or negative interpretation that people have regarding a phrase or a conversation, whereas intonation is the strength of the voice that a person gives to a phrase while speaking. In the present study, we used the tool *Sentistrength*^1^ for measuring the text polarity and pointwise mutual information (*PMI*) to classify phrases as either “excellent” or “poor” when they are being applied to the semantic orientation of a phrase (SO) (Kharrazi and Fath, 2016). For this reason, Google^2^ API phrases were used. Moreover, the semantic orientation functions with opinions that people have about words or phrases in a predetermined context. In our study, these series of opinions (*Excellent-positive, Neutral, Poor-Negative*) given by people were considered in order to obtain polarity. We processed the conversations in phrases to obtain polarity, i.e., we worked with specialized dictionaries that identify phrases in a single context. We also used the semantic orientation to classify the phrases in different contexts, because this process is based on the point of view many people. The voice analysis was based on the classification of tone and the implementation of Real-Time Emotion Recognition from Speech, verified with *Polish Emotional Speech*^3^. A voice emotion may be compared with the polarity and *PMI* since they are classified into positive–excellent and negative-poor bands. The basis of interviews was obtained from Charlotte^4^ (Control Group, healthy old patients), where the text and audio of each interview were found. Here, there was no high variability (dissociation) between the values obtained and the tools used. It was also tested in early stage AD. As a result, the dissociation between polarity, semantic orientation, and intonation was obtained. We carried out a chi-squared analysis with the aim of verifying whether there is a relationship between the variables and class. The values obtained from the experiments produced the data file for classifier algorithms for cognitively normal people and those with AD. In order to better explain our proposal, the following section describes the related research with the proposed methodology. Here, we describe the research topic to identify the relationship between polarity and semantic orientation, followed by the experimentation, conclusions, and future work on this study.

## State of the art

Frameworks have already been developed in order to analyze the emotional change in patients, i.e., with the purpose of identifying emotional disorders (Kneebone, 2016). Classifiers of depression and anxiety help us to observe the dissociations in written and oral therapies in patients with brain damage caused by mental illnesses (Waldron et al., 2012). Within the context of dissociation and emotion, techniques have shown the presence of primary aphasia in patients while they were observing abstract art. It concludes that the dissociation between emotion and art can affect the generic decoding mechanisms as well as the esthetic processing in patients with neurodegenerative diseases (Cohen et al., 2016).

Semantic orientation was used particularly in the polarity analysis. The analysis of the polarity letter dashes allows us to identify the lack of hilarity and semantic connections in a text (Onofri et al., 2015), as well as the progression of mental illnesses (Blackburn and Reuber, 2015). Emotional information is connected with the person's emotion, but it is not presented when there is brain damage and when there is a dissociation between information and emotion. Peretz and Gagnon (1999), who focused on the emotional analysis for the identification of melodies, concluded that a patient has dissociations between the identification of known and unknown melodies tied to the emotion. Bipolar disorder has been classified with a high rate of certainty by analysis tools provided by smartphones. These are related to the voice and accelerometers, and help to identify the moments of bipolarity and the emotional dissociation in different contexts of a patient's daily life (Maxhhuni et al., 2016). In the early stages of AD, patients have dissociations between language and body expression related to their personality, which is one of the main characteristics of the disease. Classifiers have been developed based on the analysis of verbal and non-verbal memory, which enables us to identify AD and to avoid confusion with other diseases, especially with frontotemporal dementia, where dissociation is a key indicator to establish differences (Baldock et al., 2016).

The dissociation between memory and context during the early stages of AD produces a deterioration pattern in the episodic memory in the short term. In fact, the semantic memory in these types of patients has a high deterioration, unlike working memory. The techniques that were used identified the loss of spontaneous memory. Semantic recognition is connected to dissociation, which is a key indicator of the clinical manifestation of the disease (Joubert et al., 2016). The semantic dissociation in people with mental illness ends when naming people and common objects, often causing *anomalies*. It is possible to eliminate the dissociation through the semantic association of known words. This semantic training approach compensates the loss caused by dissociation. Therefore, it prevents the disease progression (Heitkamp et al., 2016; Reilly, 2016). The degradation of the semantic representation is determined by the analysis of the text that is written by patients. While the mental illness progresses, the semantic paraphasia and superficial dysgraphia produce semantic dissociation as well as errors both in morphology and in syntax. For the more severe neurocognitive problems, probable AD is diagnosed if the following evidence is found: Evidence of a genetic mutation related to AD in the patient's family history or in genetic tests; Clear evidence of a loss of memory and reduced learning ability, and at least one other cognitive domain (based on detailed anamnesis or neuropsychological series tests); Progressive, gradual, and constant decline of cognitive ability without prolonged periods or “plateaus.”

The techniques identified the degradation of the semantic representations by associating them with the dissociation in the loss of vocabulary and their relationship with the objects in context. The dissociation between the cognitive elements and emotions is an indicator of mental illness when working with empathy and cognitive control. The result is that the dissociation produces changes in social behavior that affects the quality life of the patient (Maurage et al., 2016).

In conclusion, the psychological investigations related to this research work identify emotional disorders by using text and voice characteristics. The analysis and psychological techniques identify the level of dissociation. The literature shows that computer algorithms work with separated variables and do not combine their potential. In this study, we recommend combining the strength of the analysis of the variables to identify the emotional dissociation between writing and tone of voice. The results will be used by classifiers to classify patients with cognitive impairment and cognitively normal people.

## Methodology

**Figure 1:**
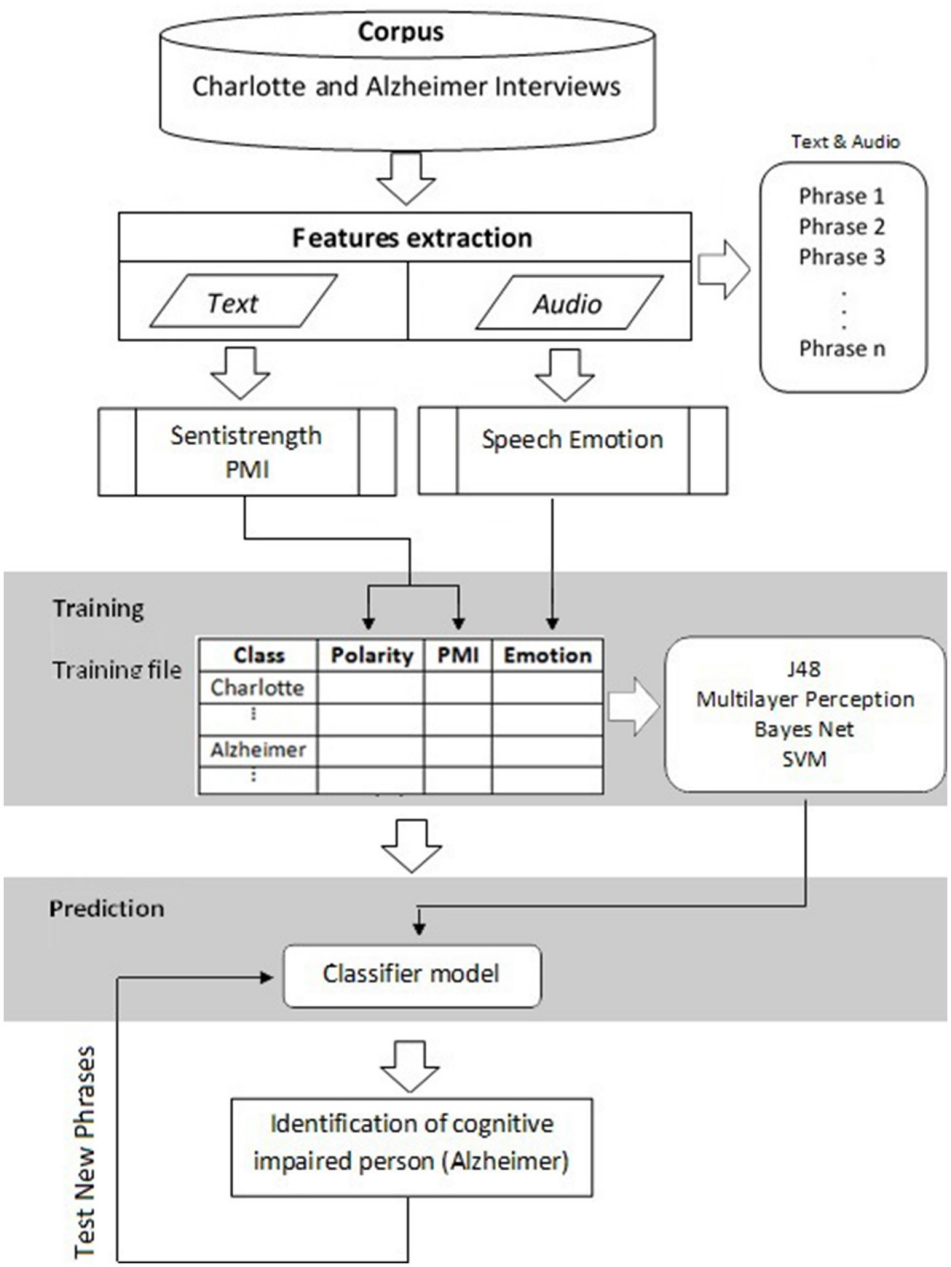
**Methodological steps for the identification of the dissociation and classification: The interviews and conversations were divided into phrases**. Each phrase was processed with polarity, *PMI*, and *Tonality*. It was then analyzed to determine any dissociation in the obtained values using Chi-squared; after verifying the dissociation, we proceeded with the classification.

Our proposal identifies the existence of a dissociation between the semantics of the text (transcription of the oral discourse), measured by polarity and semantic orientation, and emotion transmitted by the tonality of voice in patients with cognitive deficiencies. For this reason, we worked with: (a) polarity of text based on dictionaries, (b) semantic orientation by means of the *PMI*, and (c) the measure of emotional expression based on tonality when saying a phrase (see Figure 1).

In the experiments, the results show that there is a lack of correlation between polarity-tonality and also between *PMI-tonality* in patients. However, in people who are cognitively normal, the correlation exists. This dissociation is best shown when using the three variables at the same time. All steps of the methodology are described below:

### Building the training file

Interviews and conversations were divided into phrases. We processed the phrases and obtained the polarity, *PMI*, and tonality (positive/negative). With the chi-square test, we calculated the correspondence between the variables and class (normal-Charlotte, disease-AD). The training of a classifier with these data will enable us to obtain a tool that distinguishes between sick and healthy people. Figure 1 shows the proposed scheme to identify dissociation and to classify people according to the values generated by the tools.

### Polarity: *Sentistrength*

Thelwall et al. (2011, 2012) successfully applied this tool to identify the polarity of the text. It is, therefore, considered appropriate to obtain the polarity of the phrases in this research. The basic elements of *Sentistrength* are listed below (Kucuktunc et al., 2012):

Dictionary. A list of words (terms) previously classified in relation to the expressed emotion. The list has 298 positive terms and 465 negative ones. A training algorithm modifies the polarity of the predetermined words.
A list of supporting words. This list contains words that either increase or reduce the polarity of the latter words. Each word increases the polarity by 1 or 2 (for example, very, very) or decreases by 1 (for example, some).
A list of denial words. It contains words that reverse the polarity of the words later (including any word of previous reinforcement). For example, if “very happy” had positive force 4, then “not very happy” would have negative force 4.

The value of the polarity, as a result of processing the text in the tool, is calculated at intervals of −1 (negative) to −5 (very negative); and 1 (positive) to 5 (very positive). You can also obtain the output as a scale (positive/negative/neutral).

### Pointwise of mutual information: *PMI*

This technique has been used successfully to identify the semantic orientation in phrases (Read, 2004; Khan et al., 2016). The result is a classification of a phrase as *excellent and poor*, i.e., it is used as an identifier of the semantic orientation of the text. With regard to polarity, the semantic orientation is defined as *excellent* when the sentence is positive, or *poor*, when the sentence is negative. *PMI* is based on the calculation of the intersection of the terms in a phrase (Liu, 2012):

$$\text{PMI }({\text{term}}_{1},{\text{term}}_{2})=\log_{2}\left(\frac{\text{Pr }({\text{term}}_{1}\wedge {\text{term}}_{2})\text{ Pr}}{\left({\text{term}}_{1})\text{ Pr }({\text{term}}_{2}\right)}\right)$$

An intersection of the terms helps us to obtain the sentence polarity. Once this indicator is obtained, it can be used to determine whether the phrase can be classified as *Excellent or Poor* by applying the formula of the semantic orientation:

SO (phrase)​=​PMI (phrase, “excellent”)−PMI (phrase, “poor”)

The above formula requires a previous classification of similar phrases to the inputted phrase. Similar phrases can be searched in the Google API^5^, to find each sentence previously classified as *Excellent or Poor.* If the phrase entry shares its terms with the phrases of the Google API, this gives it its class.

### Tone of voice: speech emotion recognition system

This tool has been chosen since it has been proven effective with 240 recordings^6^ for five different types of emotions: Joy, boredom, anger, sadness, and neutral. The Speech Emotion Recognition algorithm trains neural^7^ networks combined with vector quantifiers and radial basis functions using tones of voice previously classified with their respective emotions (Palo et al., 2016). In order to obtain the emotion of the tonality that is inputted by the algorithm, a coefficient prediction and time-frequency are used in order to relate the tonality of input with the tones of the neural network training. The result is the emotion that corresponds to the key of the uploaded voice. Tonality of input corresponds to the raised voice key.

### Dissociation analysis: chi-square

The Chi-square calculation helps to corroborate the H1 hypothesis: There is a relationship between polarity-tone and healthy people. In this study of H1, there is a relationship between semantic-tone orientation and healthy people (*P*-value was always less than 0.05, i.e., with 2 degrees of freedom for AD with alterations of behavior). Based on this theory, one can conclude that the polarity variables, semantic orientation, and tonality of voice can be used to form part of the training of the classifiers. The results show that *SentiStrength* and Tonality are related with a class, just as *PMI* and Tonality are, which enables us to carry out a classification. The opposite occurs when the chi-square test for sick patients is applied in which H0 is proved, which allow continuing with the classifiers. With the aim of confirming the experimental results even further, we proceeded to classify one and then two variables. However, the results were not promising. The converse occurred when we classified all the variables.

### Classifiers

J48 classifiers were used along with Multilayer Perceptron, Bayes Net, and SVM because they have produced considerable success in the classification of experiments in neuroscience, and in the classification of patients with cognitive impairment vs. cognitively healthy patients (Iyer et al., 2012; Matoug et al., 2012; Shependom et al., 2013; Segovia et al., 2015). In our experiments, we initially differentiated a series of training data from testing data. However, due to the reduced number of cases available, especially of early AD conversations, and in order to establish a test file, we performed a cross-validation (Lehmann et al., 2007) test.

In the following section, we describe the experiment used for the implementation of the described methodology. The results show that the studied variables help in classifying the available cases that have a level of success, which enabled us to confirm the proposed hypothesis.

### Experimentation

The experiment was performed with two sets of data: Charlotte (with free distribution) and AD patients, which were obtained by our own team. The description of the data for the experiment is presented below:

#### Data description

##### Charlotte^8^

A wide collection of interviews exist to preserve oral memories of elderly people, especially those over 65 who were encouraged to share their memories and stories. This collection also includes interviews conducted in 1960 and 2000. This collection includes topics such as the historic preservation and reminiscences of the Second World War. In this study, Charlotte is the control group where we used 60 interviews (10 min per conversation). Each interview was divided into text and audio phrases. For this experiment, 300 phrases for each interview were taken. This was the average number of phrases to complete the entire conversation, implying three phrases per minute, thus indicating the time for the interviewee to interact with the interviewer.

##### Alzheimer's disease collection

This collection was obtained by using past videos of conversations about free topics. Patients with early stage AD were included. They came from different parts of Ecuador. Generally, the conversations recorded experiences about their life and their work. We worked with 22 interviews (10 min per conversation). Each conversation was divided into text and audio phrases, and 300 phrases per interview were used for this experiment. This was the average number of phrases to complete the entire conversation.

The phrases (Charlotte and Alzheimer) were processed with *Sentistrength, PMI*, and Speech Emotion Recognition. The results were placed in a training file.

#### Chi-square test

The results from Table 1 show a relationship between the variables taken from both pairs.

**Table 1:** **Chi-square: Healthy vs. AD**.

**Patients**	***SentiStrength* Tonality**	***PMI*-Tonality**
Healthy	4,3 E–15	2,8 E–S
Alzheimer	0.41	0.07

Table 1 shows the expected variables obtained for the Chi-square test. We then proceeded to analyze 206 phrases with the aim of corroborating whether there is a relationship between the values of *Sentistrength, Tonality*, and Class. The results showed that H1 can be corroborated (*P* < 0.05). The same occurred with *PMI*-*Tonality-*Class for a Chi-square value of 2,85E–5. Results for the chi-square test for AD showed that *Sentistrength*–*Tonality* was 0.41 and 0.07 for *PMI-Tonality*, which meant that H0 was accepted. This analysis allows us to continue with the classification process.

#### Classification

The results from Table 1 proves H0 (healthy patients) and H1 (Alzheimer patients), which is why it is possible to create a training card that helps classify the individuals in a study.

Table 2 shows an example of the values obtained by the tools. The results were as follows: The Polarity values were in the range of −5 to 5, while the values of *PMI* were represented with 1 when the emotional state is excellent, 0 if the value is poor, and −1 if the result of poor has an extremely negative value. The value for the tonality of the emotion is 1 if the emotion is positive, −1 if it is negative, and 0 if it is neutral. The values for the AD class almost matched, and also changed from one sentence to another. If we compared them with the values of Charlotte's phrases, it corroborates the hypothesis that normal people do not have the dissociation, whereas patients with early AD do. The training file was made up of Charlotte and Alzheimer's collections, which were used to train and carry out the test of 4 classifiers. The method that was selected was cross-validated with 10-folds, as proposed in (Srivastava et al., 2016) for the classification of the degeneration in patients with AD.

**Table 2:** **Example of the training file**.

**CLASS**	**POLARITY**	***PMI***	**EMOTION**
Charlotte	3	1	1
Charlotte	−5	−1	−1
Charlotte	−5	−1	−1
Charlotte	−3	−1	−1
Charlotte	−1	−1	−1
Charlotte	−4	−1	−1
Charlotte	−3	1	1
Charlotte	5	1	1
Alzheimer	5	−1	−1
Alzheimer	−3	1	0
Alzheimer	5	0	1
Alzheimer	5	1	1
Alzheimer	−1	1	1
Alzheimer	−1	0	1
Alzheimer	3	1	−1
Alzheimer	5	0	0
Alzheimer	3	1	−1
Alzheimer	−4	1	1
Alzheimer	−5	0	1
Alzheimer	5	1	−1

Tables 3–5 show the results of the classification with a single variable. These demonstrate that individually they are not useful for estimating the classification, the opposite happens when variables are combined. Tables 6–7 show that the variables improve when they are combined—reaching an F1 Score of 0.67 and ROC = 0.94 (bold value). The test was carried out with the three variables together. Table 8 shows BayesNet as a probable indicator of the onset of AD, because their precision and recall values have a certain equilibrium with the F1 Score (0.90) and ROC (0.89) (bold value).

**Table 3:** **Classification with *Sentistrength***.

**Algorithm**	**Precision**	**Recall**	**F1 score**	**ROC**
J48	0.63	0.48	0.54	0.39
Multilayer Perception	0.5	0.18	0.26	0.36
Bayes net	0.45	0.28	0.35	0.62
SVM	0.51	0.25	0.33	0.32

**Table 4:** **Classification with *Tonality***.

**Algorithm**	**Precision**	**Recall**	**F1 score**	**ROC**
J48	0.58	0.3	0.39	0.32
Multilayer Perception	0.51	0.25	0.33	0.39
Bayes net	0.56	0.36	0.44	0.47
SVM	0.47	0.28	0.35	0.50

**Table 5:** **Classification with *PMI***.

**Algorithm**	**Precision**	**Recall**	**F1 score**	**ROC**
J48	0.57	0.4	0.47	0.96
Multilayer Perception	0.62	0.51	0.56	0.52
Bayes net	0.68	0.43	0.53	0.94
SVM	0.69	0.68	0.68	0.83

**Table 6:** ***SentiStrength-Tonality***.

**Algorithm**	**Precision**	**Recall**	**F1 Score**	**ROC**
J48	0.73	0.46	0.57	0.82
Multilayer Perception	0.68	0.68	0.68	0.65
Bayes net	0.75	0.61	**0**.**67**	**0**.**93**
SVM	0.52	0.36	0.43	0.85

**Table 7:** ***PMI-Tonality***.

**Algorithm**	**Precision**	**Recall**	**F1 Score**	**ROC**
J48	0.57	0.46	0.57	0.82
Multilayer Perception	0.62	0.51	0.56	0.52
Bayes net	0.68	0.43	0.53	0.94
SVM	0.69	0.68	0.68	0.83

**Table 8:** ***SentiStrength-PMI-Tonality***.

**Algorithm**	**Precision**	**Recall**	**F1 Score**	**ROC**
J48	0.76	0.58	0.66	0.88
Multilayer Perception	0.69	0.64	0.66	0.78
Bayes net	0.88	0.93	**0.90**	**0.89**
SVM	0.71	0.58	0.64	0.88

**Figure 2:**
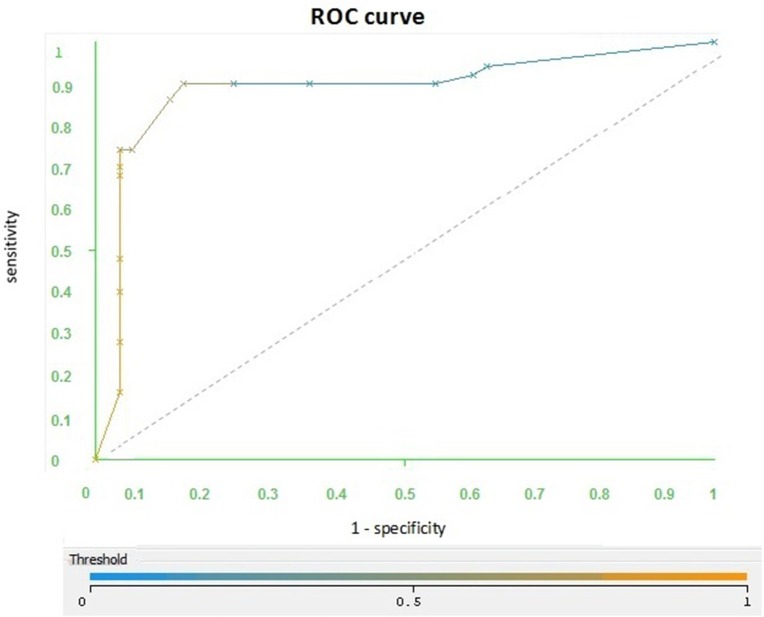
**ROC curve based on the cross validation: The curve is located close to the upper left point and AUC = 0.89**. In order to check the classifier‘s efficiency, we proceeded to build a ROC curve with a test file.

In order to check the predictive ability of BayesNet classifier, a ROC curve was built based on the training and tests files. With the training files, we obtained the cross-validation with 10-folds, as shown in Figure 2.

**Figure 3:**
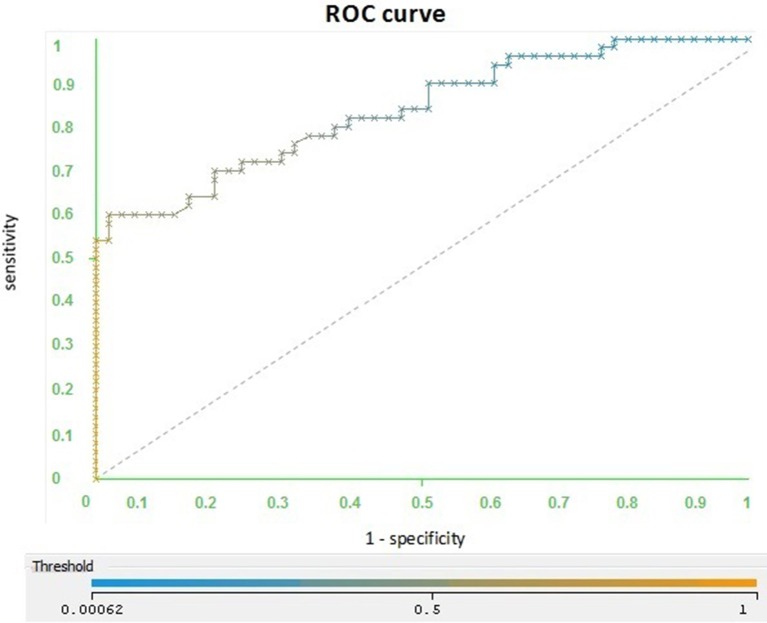
**ROC curve built from the test: The ROC curve that was obtained by separating a file test with a case battery differentiated by the training**.

We worked with a file consisting of 100 phrases for AD and 100 phrases for Charlotte. The curve (Figure 3) is located on the diagonal indicator near the left top point. The area under the curve is 0.74, which is the same value as in the cross-validation, which is 0.7.

## Conclusions and further research work

In this study, we present a methodology that aims to identify levels of dissociation among polarity, point mutual information, and emotion in the tone of voice of AD patients. This was done to obtain a simple and economic mechanism, which can help to make a diagnosis and identify an early cognitive disease, in our case, early AD. The theory is that in cognitively normal people, dissociation does not exist. We worked with phrases from the control group and patients with AD (early stage). The input file characteristics showed dissociation in patients with AD, while the converse happens in the control group. In this way, the input file was submitted to the classifiers to obtain Precision, Recall, F1 scores, and ROC (Table 8). This was compared with those obtained by other methods (Ford et al., 2016; Srivastava et al., 2016). In the study by Martínez-Murcia et al. (2012) using neuroimaging, the area under the curve was 0.92, which indicates that it is a determining classifier of the disease presence. Our results show that we have a possible indicator of early AD, due to areas of 0.89 and 0.7 obtained, which is considered acceptable for an ROC curve. The results are also comparable to those obtained by Guerrero et al. (2016) with 91 and 94% precision for detecting AD, while our study obtained a precision of 88%. These studies can be compared with early indicators or screening of the likely onset of AD. In this study, the Bayes.net algorithm obtained better results perhaps because it maintains the probabilistic management of the pattern and error. This information enables us to conclude that the method based on dissociation can be used for the identification of patients, although more tests with a higher number of conversations with patients with different cognitive impairments are necessary. In addition, the dissociations are displayed in phrases of patients with early stage AD. Apparently, they change polarity several times in the same paragraph. This can also be a sign of the disease. This probability appeared while the experiment was being developed. Although ours is a preliminary study, the next step is to identify different sources of data and characteristics of patients. Apart from providing a follow-up of the interviewees, with the aim of identifying the illness at an early stage, one can study the utility of the classifier for tests or initial filtering. We aim to analyze a higher number of conversations in a future analysis in order to accept or reject a likely indicator of AD during its early stages.

## Ethics statement

This study has been validated by the Bioethics' Committee at the Technical University of Ambato, based on the following reasons: (a) The data collected are purely observational (non-invasive and non-interactive; (b) Charlotte involves information freely available in the public domain (http://nsv.uncc.edu/nsv/narratives); (c) We use Alzheimer's disease data with anonymized records, along with a signed informed consent form from patients, family, and caregivers.

## Author contributions

SA: (a) Art State: Theoritical fundamentals of cognitive impairements; (b) Experimentation with Alzheimer's Disease; (c) Write the paper; (d) Conclusions structure. RM: (a) Introduction redaction; (b) Experimentation: Review and experiment supervised. HG: (a) Methodology structure; (b) Experimentation: Control group (Charlotte). VH: (a) Statistical and experimentation. Ethical Committee. JB: (a) Introduction Philosophy. JS: (a) Experiments and paper format. Ethical Committee. JM: (a) Experiments and paper format. Ethical Committee.

### Conflict of interest statement

The authors declare that the research was conducted in the absence of any commercial or financial relationships that could be construed as a potential conflict of interest.

## Footnotes

^1^http://sentistrength.wlv.ac.uk/

^2^https://developers.google.com/speed/libraries/

^3^https://www.linkedin.com/pulse/speech-emotion-recognition-system-matlab-source-code-luigi-rosa

^4^http://www.scit.wlv.ac.uk/~cm1993/papers/SentiStrengthPreprint.doc

^5^https://developers.google.com/speed/libraries/

^6^https://www.linkedin.com/pulse/speech-emotion-recognition-system-matlab-source-code-luigi-rosa

^7^http://www.advancedsourcecode.com/speechemotion.asp

^8^http://newsouthvoices.uncc.edu/nsv/southernpiedmont
